# 糖皮质激素在血液病患者粒细胞缺乏伴PSI评分中高危肺炎中的治疗效果

**DOI:** 10.3760/cma.j.cn121090-20240624-00234

**Published:** 2024-11

**Authors:** 述芳 薛, 金华 任, 力津 陈, 小琴 赵, 婷 杨, 建达 胡

**Affiliations:** 1 福建医科大学附属协和医院血液科，福建省血液病重点实验室，福建省血液病研究所，福州 350001 Fujian Institute of Hematology, Fujian Medical University Union Hospital, Fuzhou 350001, China; 2 福建省儿童医院血液肿瘤科，福州 350014 Department of Hematology-Oncology, Fujian Children's Hospital, Fuzhou 350014, China; 3 福建医科大学附属第一医院滨海院区、国家区域医疗中心血液科&血液移植中心，福州 350209 Department of Hematology, National Regional Medical Center, Binhai Campus of the First Affiliated Hospital, Fujian Medical University, Fuzhou 350209, China; 4 福建医科大学附属第一医院血液科，福州 350005 Department of Hematology, the First Affiliated Hospital, Fujian Medical University, Fuzhou 350005, China; 5 福建医科大学精准医学研究院，福州 350122 Institute of Precision Medicine, Fujian Medical University, Fuzhou 350122, China; 6 福建医科大学附属泉州第一医院肿瘤内科，泉州 362000 Department of Oncology, Quanzhou First Hospital Affiliated to Fujian Medical University, Quanzhou 362000, China; 7 福建医科大学附属协和医院平潭医院血液科，平潭 350128 Department of Hematology, Fujian Medical University Union Hospital, Pingtan Hospital, Pingtan 350128, China; 8 福建医科大学附属第二医院，泉州 362000 Department of Hematology, the Second Affiliated Hospital, Fujian Medical University, Quanzhou 362000, China

**Keywords:** 糖皮质激素, 粒细胞缺乏, 肺炎严重指数, 重症肺炎, Glucocorticoids, Neutropenia, Pneumonia severity index, Severe pneumonia

## Abstract

**目的:**

探索糖皮质激素在血液病患者粒细胞缺乏（粒缺）合并肺炎严重指数（pneumonia severity index，PSI）中高危肺炎中的治疗价值。

**方法:**

回顾性分析2016年10月1日至2018年12月31日福建医科大学附属协和医院血液科收治的粒缺合并PSI中高危肺炎的534例血液病患者资料，利用倾向性评分（PSM）调整激素组与非激素组之间基础资料的差异，比较两组患者治疗过程中炎症因子的变化，治疗失败率、死亡率、到达临床稳定状态时间、抗菌药物使用天数及不良反应发生率。

**结果:**

176例患者接受了激素治疗，而358例患者未使用激素。激素组患者炎症因子水平、合并症比例及PSI评分更高。PSM共匹配125对病例。匹配后激素组和非激素组之间的合并症差异减小，但激素组的炎症因子水平仍然较高，接受激素治疗的患者病情较重，而在后续的治疗过程中炎症因子水平的下降更为显著。激素组晚期治疗失败率高于非激素组（39.2％对24.8％，*P*＝0.015），但主要体现在影像学进展，而呼吸衰竭、机械通气、脓毒性休克等严重并发症的发生率差异无统计学意义。Logistic回归分析显示，糖皮质激素可降低治疗失败率（*OR*＝0.367，95％*CI* 0.165～0.818，*P*＝0.014）。PSI评分高增加治疗失败率（*OR*＝1.028，95％*CI* 1.007～1.049，*P*＝0.008）。激素组与非激素组30 d死亡率差异无统计学意义（8.0％对7.2％，*P*＝0.811）。PSI评分是30 d死亡的危险因素（*OR*＝1.077，95％*CI* 1.032～1.123，*P*＝0.001）。激素组PSI Ⅴ级患者30 d生存率与PSI Ⅳ级患者比较差异无统计学意义［（87.8±5.1）％对（94.0±2.6）％，*P*＝0.216］。糖皮质激素并不增加血糖升高、消化道出血和30 d内再感染发生率。

**结论:**

糖皮质激素在血液病患者粒缺合并PSI中高危肺炎的治疗中有助于控制炎症因子水平，可降低PSI Ⅴ级患者治疗失败率及死亡率。

## Abstract (en)

**Objective:**

This study aimed to investigate the clinical value of glucocorticoids in patients with neutropenic severe pneumonia at moderate to high risk according to the Pneumonia Severity Index（PSI）in patients with hematologic diseases.

**Methods:**

Clinical data were collected from 534 patients at the Fujian Medical University Union Hospital from October 2016 to December 2018. We evaluated the changes in inflammatory cytokines, treatment failure, in-hospital mortality, and other outcomes, adjusting for potential confounders through propensity score matching.

**Results:**

Patients were categorized into glucocorticoids（*n*＝176）and control（*n*＝358）groups. The glucocorticoid group demonstrated higher levels of C-reactive protein, procalcitonin, and interleukin-6, alongside higher PSI scores. The differences in comorbidities diminished, except for inflammatory cytokine levels, with a notable reduction in inflammatory cytokines observed in the glucocorticoid group, after matching 125 pairs based on propensity scores. Late treatment failure was more prevalent in the glucocorticoid group（39.2％ *vs* 24.8％, *P*＝0.015）, but this was primarily caused by radiographic progression. The incidences of respiratory failure, mechanical ventilation, and septic shock were similar between the groups. Logistic regression analyses revealed that glucocorticoids reduced the risk of treatment failure（*OR*＝0.367, 95％ *CI* 0.165–0.818, *P*＝0.014）. The 30-day in-hospital mortality rates were comparable（8.0％ in glucocorticoids *vs* 7.2％ in controls, *P*＝0.811）, with indications that glucocorticoids may exert a protective effect on mortality. The PSI score was determined as the sole independent risk factor for 30-day in-hospital mortality（*OR*＝1.077, 95％ *CI* 1.032–1.123, *P*＝0.001）. No evidence indicated that glucocorticoids increased the incidence of hyperglycemia, gastrointestinal bleeding, or 30-day infection recurrence.

**Conclusion:**

Glucocorticoids reduce inflammatory cytokine levels and are potentially related to decreased treatment failure and mortality in patients with neutropenic pneumonia classified as PSI Ⅳ and Ⅴ among hematological patients.

近年来，血液病的治疗取得了显著进展，但治疗后的粒细胞缺乏（粒缺）伴感染对患者构成严重威胁，肺部感染尤为常见且严重[1]–[2]。接受诱导化疗的白血病患者中，肺部感染的发生率为13％～31％，死亡率高达25％～45％[3]–[4]。抗菌药物是粒缺合并肺炎的主要治疗手段，但全身炎症反应可能导致器官功能衰竭甚至死亡[5]–[6]。临床医师也在尝试使用糖皮质激素等抗炎药物辅助治疗。炎症因子如IL-6、TNF-α等可激活下丘脑-垂体-肾上腺轴，促进肾上腺皮质激素的释放。糖皮质激素可作用于AP-1及NF-κB等转录因子及细胞信号传导通路等环节，下调炎症因子如IL-1、IL-2、IL-3、IL-6、IL-8、TNF-α等而上调抗炎因子如IL-10等的水平[7]–[8]。然而，30％重症患者及50％～60％脓毒性休克患者可合并肾上腺皮质功能不全，这部分患者对缩血管药物反应更差，死亡率更高[9]–[10]。同时，糖皮质激素在肺部发挥以下3个方面的作用：抑制呼吸道上皮细胞NF-κB的表达、抑制黏液腺的分泌、结合平滑肌β_2_受体而舒张支气管[8]。在肺炎治疗中使用糖皮质激素的争议由来已久。既往研究认为糖皮质激素的使用可能有助于控制临床症状以及减少并发症，但是否降低病死率仍不明确，部分研究认为只有重症肺炎患者有所获益[11]–[14]。目前大部分肺炎的治疗指南中尚未推荐常规使用糖皮质激素，但有部分推荐在合并脓毒血症、脓毒性休克患者中可使用糖皮质激素，其中部分考虑与肾上腺皮质功能不全有关[15]。

对于免疫抑制患者，重症肺炎的诊断仍无统一标准。肺炎严重指数（pneumonia severity index，PSI）由Fine等[16]提出，包括人口学因素、合并的基础疾病、体格检查以及辅助检查等20个因素，通过积分划分为5个级别，社区获得性肺炎（community-acquired pneumonia, CAP）死亡率随着PSI分级的增高而增高[16]。既往研究通过对比PSI与其他多项评分系统，发现对于免疫抑制患者，PSI同样对预测30 d死亡具有高敏感性[17]–[18]。为进一步探索糖皮质激素的临床价值，本文回顾性分析糖皮质激素治疗血液病粒缺合并PSI中高危（评分>90分）肺炎患者的临床资料如下。

## 对象与方法

1. 研究对象：回顾性分析2016年10月至2018年12月期间福建医科大学附属协和医院血液科收治的符合以下条件的患者临床资料：①年龄≥16周岁；②ANC<0.5×10^9^/L；③新近出现发热、咳嗽、咳痰等肺炎相关的症状；④影像学提示新出现的炎性渗出等改变；⑤PSI评分在Ⅳ级及以上。排除标准：①此次治疗前因其他疾病需要使用糖皮质激素；②治疗中因各种原因中断治疗或不配合治疗；③合并其他严重或可能危及生命的疾病状态；④住院时间不超过48 h。

收集资料包括基础疾病、生命体征、实验室指标、影像学检查、合并症、疗效指标（并发症、治疗失败率、死亡率、到达临床稳定状态时间、住院天数等）、不良反应（血糖升高、消化道出血、30 d内再感染发生率）。

2. 相关定义：累计静脉使用糖皮质激素时间≥3 d定义为激素组，其他为非激素组。脓毒性休克：感染患者当脓毒症相关序贯器官衰竭（sequential organ failure assessment, SOFA）评分较基线上升≥2分的基础上，出现持续性低血压，在充分液体复苏后仍需血管活性药来维持平均动脉压≥65 mmHg（1 mmHg＝0.133 kPa）以及血乳酸水平≥2 mmol/L[19]。呼吸衰竭：动脉血氧分压（PaO_2_）/吸入氧分数（FiO_2_）<200 mmHg，且呼吸频率>30次/min[20]。治疗失败：开始抗菌药物治疗后出现脓毒性休克、死亡、呼吸衰竭、影像学进展（较前增加50％）。早期治疗失败指72 h内出现的上述情况，晚期治疗失败指72 h后出现[19]。血糖升高：指入院空腹血糖在7.0 mmol/L以下，治疗后复查空腹血糖大于7.0 mmol/L。临床稳定状态：持续24 h及以上的稳定生命体征［体温≤38 °C；心率≤100次/min；呼吸≤24次/min；收缩压≥90 mmHg（高血压者≥100 mmHg）；神志、进食正常］[21]。

3. 统计学处理：使用SPSS 24.0软件进行统计分析。采用1∶1倾向性评分匹配（PSM）方法，旨在减少组间差异可能产生的混杂影响，纳入年龄、是否移植、降钙素原（PCT）、胸腔积液、PSI等指标进行倾向得分计算，使用最近邻匹配方法，以卡钳值不超过0.005的标准进行匹配。计数资料用百分比表示，组间比较用*χ*²检验或Fisher精确概率法；计量资料符合正态分布的用*x*±*s*表示，组间比较采用独立样本*t*检验；非正态分布资料用*M*（*IQR*）表示，组间比较采用Mann-Whitney *U*检验。糖皮质激素对治疗失败率和死亡率的影响通过二分类Logistic回归分析计算*OR*及其95％*CI*。采用Kaplan-Meier曲线绘制生存曲线图分析治疗后30 d生存情况，Log-rank检验进行组间比较。双侧*P*<0.05为差异有统计学意义。

## 结果

1. 病例资料：共纳入患者534例，其中非激素组358例，激素组176例。使用的糖皮质激素包括甲泼尼龙（111例，63.1％）、地塞米松（19例，10.8％）、甲泼尼龙联合地塞米松（46例，26.1％）。甲泼尼龙每日用量为0.5～2 mg/kg，地塞米松每日用量为3～15 mg。激素中位使用时间为8（4～12）d。其中54例（10.1％）在抗菌药物治疗后24 h内给予糖皮质激素。

两组患者在性别、血液病及其他基础病方面差异无统计学意义。PSM前，激素组的PCT、C反应蛋白（CRP）、IL-6等炎症因子水平较非激素组高，PaO_2_较低。病原学检查共检出117株菌株，其中嗜麦芽窄食单胞菌（22株）、铜绿假单胞菌（20株）、鲍曼不动杆菌（15株）、肺炎克雷伯菌（15株）最为常见。影像学检查显示，激素组患者胸腔积液>1 cm比例更高。合并其他部位的感染以口腔感染（18.7％）、血流感染（16.1％）、肠道感染（13.5％）为主。总体上，激素组患者病情更重，PSI评分显著高于非激素组（130.84±28.76对114.20±20.15，*P*<0.001），PSI Ⅴ级患者比例也更高（43.2％对17.3％，*P*<0.001）。PSM共匹配125对病例。PSI差异得到控制，但PaO_2_差异仍具有统计学意义（表1）。

**表1:** 倾向性评分匹配（PSM）前后血液病患者是否应用激素治疗组基线临床特征比较

临床资料	PSM前			PSM后		
	非激素组（358例）	激素组（176例）	*P*值	非激素组（125例）	激素组（125例）	*P*值
年龄［岁，*M*（*IQR*）］	55（48，62）	54（44，61）	0.038	54（40，62）	55（46，62）	0.658
男性［例（％）］	240（67.0）	122（69.0）	0.596	87（70.0）	80（64.0）	0.347
基础疾病［例（％）］						
充血性心衰	5（1.4）	3（1.7）	0.722	3（2.4）	1（0.8）	0.622
肾病	11（3.1）	11（6.3）	0.082	4（3.2）	4（3.2）	>0.999
肝病	56（15.6）	31（17.6）	0.562	26（20.8）	16（12.8）	0.091
COPD	1（0.3）	0（0）	>0.999	1（0.8）	0（0）	>0.999
糖尿病	55（15.4）	22（12.5）	0.376	23（18.4）	19（15.2）	0.499
血液病类型［例（％）］			0.175			0.240
AML	195（54.5）	94（53.4）		67（53.6）	66（52.8）	
ALL	63（17.6）	21（11.9）		23（18.4）	11（8.8）	
MDS	31（8.7）	15（8.5）		10（8.0）	13（10.4）	
MM	9（2.5）	11（6.3）		5（4.0）	5（4.0）	
NHL	47（13.1）	26（14.8）		14（11.2）	22（17.6）	
其他	13（3.6）	9（5.1）		6（4.8）	8（6.4）	
化疗［例（％）］	318（88.8）	151（85.8）	0.314	113（90.4）	108（86.4）	0.323
移植［例（％）］	3（0.8）	10（5.7）	0.001	1（0.8）	3（2.4）	0.622
实验室检查						
PCT［µg/L，*M*（*IQR*）］	0.272（0.151，0.779）	0.405（0.185，1.465）	0.017	0.324（0.169，0.989）	0.408（0.185，1.485）	0.428
CRP［mg/L，*M*（*IQR*）］	84.1（35.4，127.0）	128.0（48.5，154.3）	0.045	85.2（43.5，136.8）	116.0（72.9，162.0）	0.066
IL-6［ng/L，*M*（*IQR*）］	67.0（40.3，179.6）	137.7（53.5，324.6）	0.055	58.9（31.1，120.7）	124.3（51.5，263.5）	0.179
血气分析						
pH［*M*（*IQR*）］	7.432（7.343，7.466）	7.430（7.362，7.472）	0.530	7.421（7.171，7.466）	7.431（7.393，7.466）	0.208
PaO_2_［mmHg，*M*（*IQR*）］	126.9（94.3，163.7）	91.65（57.8，129.9）	0.019	132.0（100.7，163.7）	103.1（58.7，149.1）	0.043
病原学检查［株（％）］						
铜绿假单胞菌	9（2.5）	11（6.3）	0.033	2（1.6）	7（5.6）	0.172
嗜麦芽窄食单胞菌	9（2.5）	13（7.4）	0.008	3（2.4）	5（4.0）	0.722
肺炎克雷伯菌	7（2.0）	8（4.5）	0.100	5（4.0）	5（4.0）	>0.999
鲍曼不动杆菌	5（1.4）	10（5.7）	0.009	1（0.8）	5（4.0）	0.213
阴沟肠杆菌	3（0.8）	5（2.8）	0.122	1（0.8）	4（3.2）	0.370
洋葱伯克霍尔德菌	2（0.6）	6（3.4）	0.018	2（1.6）	3（2.4）	>0.999
其他	17（4.7）	12（6.8）	0.321	7（5.6）	8（6.4）	0.790
肺部CT						
多肺叶浸润［例（％）］	348（97.2）	175（99.4）	0.111	118（94.4）	124（99.2）	0.066
胸腔积液>1 cm［例（％）］	94（26.3）	72（40.9）	0.001	46（36.8）	44（35.2）	0.792
PSI评分（分，*x*±*s*）	114.20±20.15	130.84±28.76	<0.001	122.39±23.97	122.70±21.56	0.894
PSI Ⅴ级［例（％）］	62（17.3）	76（43.2）	<0.001	38（30.4）	41（32.8）	0.683
合并其他感染［例（％）］						
血流感染	46（12.8）	40（22.7）	0.004	20（16.0）	20（16.0）	>0.999
肠道感染	42（11.7）	30（17.0）	0.091	24（19.2）	15（12.0）	0.117
口腔感染	66（18.4）	34（19.3）	0.806	25（20.0）	23（18.4）	0.748
肛周感染	20（5.6）	12（6.8）	0.573	9（7.2）	6（4.8）	0.424
皮肤软组织感染	28（7.8）	15（8.5）	0.779	10（8.0）	12（9.6）	0.655
尿路感染	3（0.8）	6（3.4）	0.065	0（0）	5（4.0）	0.060

**注** COPD：慢性阻塞性肺病；AML：急性髓系白血病；ALL：急性淋巴细胞白血病；MDS：骨髓增生异常综合征；MM：多发性骨髓瘤；NHL：非霍奇金淋巴瘤；PCT：降钙素原；CRP：C反应蛋白；pH：酸碱度；PaO_2_：动脉血氧分压；PSI：肺炎严重指数

2. 并发症：治疗中出现的并发症根据发生率从高到低依次为脓毒性休克（14.8％）、急性心功能不全（7.9％）、急性呼吸衰竭（5.6％）。PSM后激素组及非激素组间上述并发症的发生率差异均无统计学意义（脓毒性休克：16.0％对18.4％，*P*＝0.615；急性心功能不全：8.8％对5.6％，*P*＝0.328；急性呼吸衰竭：7.2％对4.8％，*P*＝0.424）。

3. 炎症因子水平变化情况：剔除抗菌药物使用24 h后给予糖皮质激素的患者，对剩余患者（激素组54例、非激素组251例）的炎症因子水平进行分析。激素组的基线PCT、CRP和IL-6水平明显高于非激素组。但激素组炎症因子水平的下降更为显著，第14天时PCT、CRP和IL-6水平均低于非激素组（图1）。

**图1:**
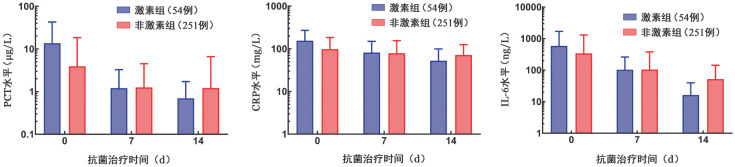
激素组与非激素组血液病患者抗菌药物治疗后炎症因子水平变化情况（剔除抗菌药物使用24 h后给予糖皮质激素的患者） **注** PCT：降钙素原；CRP：C反应蛋白

4. 治疗失败情况：共147例（27.5％）治疗失败，其中早期治疗失败5例（0.9％），晚期治疗失败142例（26.6％）。PSM后，激素组晚期治疗失败率高于非激素组（39.2％对24.8％，*P*＝0.015），但主要因影像学进展（29.6％对14.4％，*P*＝0.004），而呼吸衰竭、机械通气、脓毒性休克及死亡组间差异无统计学意义（表2）。多因素Logistic回归分析显示，糖皮质激素可降低治疗失败率（*OR*＝0.367，95％*CI* 0.165～0.818，*P*＝0.014），而PSI评分高（*OR*＝1.028，95％*CI* 1.007～1.049，*P*＝0.008）及胸腔积液（*OR*＝3.235，95％*CI* 1.404～7.452，*P*＝0.006）则增加治疗失败率。

**表2:** 倾向性评分匹配（PSM）前后血液病患者是否应用激素治疗的临床结局比较［例（％）］

临床结局	PSM前			PSM后		
	非激素组（358例）	激素组（176例）	*P*值	非激素组（125例）	激素组（125例）	*P*值
早期失败	1（0.3）	4（2.3）	0.043	1（0.8）	3（2.4）	0.622
休克	1（0.3）	2（1.1）	0.254	1（0.8）	2（1.6）	>0.999
机械通气	0（0）	2（1.1）	0.108	0（0）	1（0.8）	>0.999
晚期失败	59（16.5）	83（47.2）	<0.001	31（24.8）	49（39.2）	0.015
影像学进展	37（10.3）	57（32.4）	<0.001	18（14.4）	37（29.6）	0.004
呼吸衰竭	8（2.2）	22（12.5）	<0.001	6（4.8）	9（7.2）	0.424
机械通气	2（0.6）	4（2.3）	0.095	1（0.8）	2（1.6）	>0.999
脓毒性休克	23（6.4）	29（16.5）	<0.001	13（10.4）	11（8.8）	0.668
死亡	15（4.2）	29（16.5）	<0.001	9（7.2）	10（8.0）	0.811
总失败	60（16.8）	87（49.4）	<0.001	32（25.6）	52（41.6）	0.007
全因死亡	15（4.2）	29（16.5）	<0.001	9（7.2）	10（8.0）	0.811

5. 生存分析：住院期间共有44例（8.2％）患者死亡。PSM后，激素组与非激素组30 d死亡率差异无统计学意义（8.0％对7.2％，*P*＝0.811）。多因素Logistic回归分析显示，只有PSI评分是30 d死亡的危险因素（*OR*＝1.077，95％*CI* 1.032～1.123，*P*＝0.001）。激素组与非激素组患者PSI分级的生存曲线见图2，非激素组PSI Ⅴ级患者30 d生存率为（78.9±6.6）％，PSI Ⅳ级患者为（98.9±1.1）％（*P*<0.001）；激素组PSI Ⅴ级患者30 d生存率为（87.8±5.1）％，PSI Ⅳ级患者为（94.0±2.6）％（*P*＝0.216），提示糖皮质激素能改善PSI Ⅴ级患者的生存情况。

**图2:**
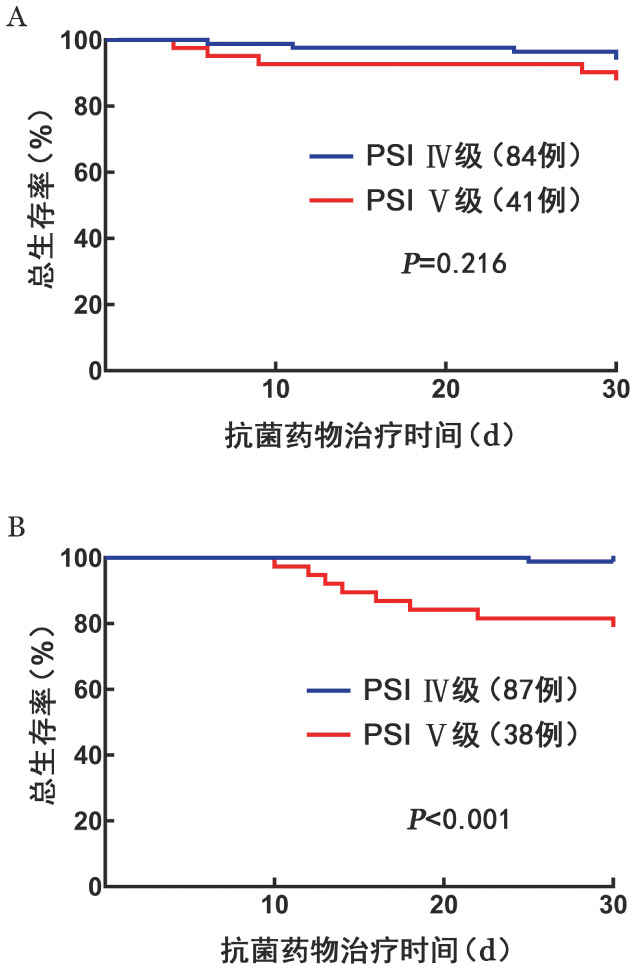
抗菌药物治疗30 d内不同肺炎严重指数（PSI）分级患者生存曲线 **A** 激素组；**B** 非激素组

6. 到达临床稳定状态及抗菌药物使用时间：所有534例患者中，到达临床稳定状态的中位时间为4（2，7）d，抗菌药物中位使用时间为18（11，25）d。PSM前后，二组患者的到达临床稳定状态中位时间均为4 d，激素组抗菌药物中位使用时间长于非激素组［22（16，28）d对15（10，24）d，*P*<0.001］。

7. 合并脓毒性休克情况：534例患者中，24例（4.5％）治疗前合并脓毒性休克，其中非激素组14例，激素组10例，激素组中9例在抗菌药物使用后24 h内开始糖皮质激素治疗。非激素组使用升压药物的时间比激素组略长［3.5（1，9）d对2.5（1.5，6）d，*P*＝0.403］，到达临床稳定状态的时间略长［6.5（1，9）d对5（2，8）d，*P*＝0.856］；激素组抗菌药物使用时间更长［25.5（12，37）d对14.5（10，22）d，*P*＝0.074］。5例（20.8％）治疗失败，激素组与非激素组之间差异无统计学意义［30.0％（3/10）对14.3％（2/14），*P*＝0.615］。激素组2例死亡，非激素组无死亡病例。

8. 不良反应：共有83例（15.5％）患者治疗后血糖升高，11例（2.1％）出现消化道出血，96例（18.0％）在治疗后30 d内再发生感染。PSM前，激素组血糖升高的发生率高于非激素组（26.7％对10.1％，*P*<0.001），PSM后差距缩小且差异无统计学意义（20.0％对15.2％，*P*＝0.319）。激素组与非激素组消化道出血发生率、治疗后30 d内再感染发生率差异均无统计学意义。

## 讨论

糖皮质激素对肺炎治疗的作用存在争议。肺炎患者异质性较大，这在一定程度上加大了研究的干扰因素，本研究将观察对象定位于粒缺合并PSI评分Ⅳ、Ⅴ级肺炎的血液病患者。本研究结果显示高PSI评分与治疗失败率及死亡率增加相关，PSI评分每增加1分，则治疗失败率及死亡率分别增加2.8％和7.7％。另外本研究采用了PSM对激素组和非激素组患者基线状态及潜在的混杂因素进行匹配，提高可比性。

炎症反应是机体重要的自我防御形式，但持续升高的炎症因子水平与严重并发症和病死率相关[5]。糖皮质激素可降低TNF-α、IL-1β及IL-6等因子水平，相关机制已明确[22]–[23]。本研究显示糖皮质激素可有效降低PSI中高危肺炎患者的炎症因子水平。Snijders等[24]的研究发现，连续7 d使用40 mg/d甲泼尼龙治疗重症肺炎，激素组在治疗早期CRP下降较快，但在第14天后CRP反而高于非激素组，故认为停药后可能出现反弹效应，但该研究中激素未减量。与Snijders等[24]研究不同之处在于，本研究中30.7％的患者逐渐减量停药，且对象为血液病中粒缺状态的重症肺炎患者，病程和激素使用时间较长，未观察到后期炎症指标的反弹情况。

在本研究中，激素组在治疗72 h后影像学进展发生率高，但严重的呼吸衰竭、机械通气和脓毒性休克发生率与非激素组差异无统计学意义。Logistic回归分析进一步提示，糖皮质激素对降低PSI中高危肺炎的患者治疗失败率有保护作用。按PSI分级进行生存分析显示，激素组PSI Ⅴ级和Ⅳ级患者的生存曲线差距缩小，提示病情更重的患者更可能从激素治疗中获益。

尽管激素组患者病情更重，但两组达到临床稳定状态的时间差异无统计学意义。糖皮质激素能有效控制体温并有利于血流动力学稳定，可能快速缓解临床症状，这可能与其强大的抗炎作用相关[25]–[26]。本研究未发现糖皮质激素缩短抗菌药物使用时间的作用。糖皮质激素通过水钠潴留增加有效循环血量，通过与血管内皮细胞的受体结合增强血管收缩力，还能改善微循环和器官灌注[27]。本研究发现在合并脓毒性休克的患者中，糖皮质激素能缩短依赖升压药物的时间和达到临床稳定状态的时间，尽管统计学差异不显著，但在临床实践中这种差异可能具有一定意义。由于样本量有限，本研究未能显示糖皮质激素对治疗失败率和死亡率的明显改善。

粒缺、粒缺持续时间、粒细胞和淋巴细胞功能、黏膜屏障损伤及既往耐药菌感染等因素，均显著影响血液病患者的感染预后[28]–[29]。此外，原发病类型也影响感染预后。例如，急性白血病患者常出现严重且持久的粒缺（超过7 d），而实体瘤患者（如淋巴瘤）则较少面临此类高危情况，而原发病的控制情况更与感染的管理密切相关[30]。糖皮质激素用于辅助抗感染治疗的效果可能因感染病原微生物的不同而有所差异，目前仍缺乏系统性研究。本研究中患者样本检出病原均为细菌，所检出病原如铜绿假单胞菌在既往研究中提示患者从激素治疗中获益[31]。病毒性肺炎以COVID-19为代表，随机临床研究表明小剂量地塞米松可降低需要呼吸支持的COVID-19肺炎患者的死亡率[32]。另外小剂量泼尼松用于辅助治疗儿童播散性念珠菌病可有效控制临床症状[33]。本研究中由于病原菌检出率低故难以进行分组分析。

本研究的主要结论包括：①糖皮质激素能有效控制炎症因子水平；②在PSI中高危肺炎患者中，糖皮质激素能降低治疗失败率和住院期间死亡率，尤其是PSI Ⅴ级患者可能更能从治疗中获益；③对于合并脓毒性休克的患者，糖皮质激素能缩短需依赖升压药物的时间和达到临床稳定状态的时间；④糖皮质激素不增加血糖升高、消化道出血及30 d内再感染的风险。

本研究的局限性包括：①回顾性观察性研究设计，尽管采用PSM提高了患者组间可比性，但无法评估部分潜在混杂因素；②对糖皮质激素的剂量、开始时间、疗程及给药方式等因素缺乏详细分析，这些可能影响结局；③未获得与肾上腺皮质功能相关的临床数据，可能未能完全识别高危患者；④由于病原学检出率不高，未能进行根据病原种类的亚组分析；⑤由于许多患者均为初发诱导化疗中出现粒缺伴感染而未能对原发病的情况进行评估；⑥粒缺持续时间、粒细胞及淋巴细胞的功能等与感染的控制密切相关的因素可能对本研究产生影响，但因回顾性研究临床资料有限，未能进行具体分析。

综上所述，糖皮质激素能有效控制粒缺合并PSI中高危肺炎的炎症反应，可能降低治疗失败率及死亡率，并且PSI Ⅴ级患者更可能在激素治疗中获益。对于合并脓毒性休克者而言，糖皮质激素有利于稳定血流动力学。治疗中短期使用糖皮质激素无明显不良反应。
